# A Smart Region-Growing Algorithm for Single-Neuron Segmentation From Confocal and 2-Photon Datasets

**DOI:** 10.3389/fninf.2020.00009

**Published:** 2020-03-17

**Authors:** Alejandro Luis Callara, Chiara Magliaro, Arti Ahluwalia, Nicola Vanello

**Affiliations:** ^1^Research Center “E. Piaggio” - University of Pisa, Pisa, Italy; ^2^Dipartimento di Ingegneria dell’Informazione, University of Pisa, Pisa, Italy

**Keywords:** neuron segmentation, confocal microscopy, 2 photon microscopy, expectation - maximization (EM) algorithm, mixture models, CLARITY

## Abstract

Accurately digitizing the brain at the micro-scale is crucial for investigating brain structure-function relationships and documenting morphological alterations due to neuropathies. Here we present a new Smart Region Growing algorithm (SmRG) for the segmentation of single neurons in their intricate 3D arrangement within the brain. Its Region Growing procedure is based on a homogeneity predicate determined by describing the pixel intensity statistics of confocal acquisitions with a mixture model, enabling an accurate reconstruction of complex 3D cellular structures from high-resolution images of neural tissue. The algorithm’s outcome is a 3D matrix of logical values identifying the voxels belonging to the segmented structure, thus providing additional useful volumetric information on neurons. To highlight the algorithm’s full potential, we compared its performance in terms of accuracy, reproducibility, precision and robustness of 3D neuron reconstructions based on microscopic data from different brain locations and imaging protocols against both manual and state-of-the-art reconstruction tools.

## Introduction

Digitizing a high-fidelity map of the neurons populating the brain is a central endeavor for neuroscience research and a crucial step for the delineation of the full Connectome (Alivisatos et al., 2012). Moreover, single-neuron reconstruction from empirical data can be used to generate models and make predictions about higher-level brain organization, as well as to study the normal development of dendritic and axonal arbors or document neuro-(patho)physiological changes (Budd et al., 2015).

Confocal and two-photon microscopy are considered the best candidates to image defined cellular populations in three-dimensional (3D) biological specimens (Wilt et al., 2009; Ntziachristos, 2010). Their imaging depth, as well as the quality of the acquired datasets can be further improved thanks to recent tissue-clearing solutions, which render brain tissue transparent to photons by reducing the source of scattering, allowing confocal acquisitions with enhanced Signal to Noise Ratios and Contrast to Noise Ratios while maintaining low laser power (Chung and Deisseroth, 2013; Richardson and Lichtman, 2015; Magliaro et al., 2016). While these technologies and protocols, combined with fluorescence-based labeling techniques, enable the imaging of the brain’s intricacies at the microscale, single-cell segmentation algorithms able to deal with these datasets are still lacking (Magliaro et al., 2019), despite targeted initiatives such as the DIADEM (DIgital reconstructions of Axonal and DEndrite Morphology) challenge in 2009–2010 (Gillette et al., 2011) and the BigNeuron project in 2015 (Peng et al., 2015). In fact, different approaches have been implemented for reaching the goal of segmentation of single cells (Acciai et al., 2016). Most of these tools reconstruct the pathway of neurite or neural processes, i.e., neuron tracing (Quan et al., 2016; Kayasandik et al., 2018) using different approaches, ranging from active contour methods (Kass et al., 1988; Wang et al., 2009; Baswaraj et al., 2012) to hierarchical pruning (Peng et al., 2011a; Xiao and Peng, 2013), in an attempt to face the a number of key challenges: (i) noisy points causing over-tracing, (ii) gaps between continuous arbors causing under-tracing, and (iii) non-smooth surfaces of the arbors violating geometric assumptions (Liu et al., 2016). Among them, machine learning approaches are widely considered as robust for neural structure segmentation in image stacks (Januszewski et al., 2018; Sakkos et al., 2018). These methods mainly consist in building a classifier able to discern between foreground and background, thanks to prior information obtained through a training dataset of manually-segmented neuron structures. However, building the training dataset is very time consuming, in particular because it needs to be fleshed out when dealing with different images (e.g., neuron types with different morphology or stacks with different background/foreground features). Finally, many tools and algorithms for neuron segmentation primarily focus on sparsely labeled data, such that their application to images (or volumes) representing densely packed neurons, typical of mammalian brains, is limited (Chothani et al., 2011; Wang et al., 2011, 2017; Peng et al., 2014; Hernandez et al., 2018).

The outcomes of neuron reconstructions are traditionally stored in a.swc file format, where spatial (i.e., *x*, *y*, and *z* coordinates) and morphological (e.g., neurite thickness) information about specific points of interest (e.g., neuron nodes) are listed. This standard describes neuron morphology with a number of structurally connected compartments (e.g., cylinders or spheres representing neuron arborization or soma, respectively), thereby neglecting the morphological and volumetric information along the neuron’s length (Magliaro et al., 2019).

Confocal and 2-photon datasets are characterized by on-plane and intra-plane pixel intensity heterogeneities, deriving from optical phenomena and the non-uniform distribution of fluorophores through the sample (Diaspro, 2001). Given these intrinsic features, a valid procedure for accurately digitizing the neural structures in the stack could be obtained by leveraging on local approaches and methods enforcing spatial constraints, such as region growing procedures (RG) (Brice and Fennema, 1970; Xiao and Peng, 2013; Acciai et al., 2016). RG is a pixel intensity-based segmentation method that identifies the foreground starting from a pixel, i.e., the seed, belonging to the foreground itself. The neighboring pixels of the seed are iteratively examined based on a predefined rule, usually a homogeneity predicate, which can be estimated locally to determine whether they should be added to the foreground or not. The performance of the procedure may be influenced by both the seed selection and the rule (Baswaraj et al., 2012). The choice of the rule may be non-trivial, in particular in view of delivering a general-purpose segmentation algorithm. Adaptive strategies based on mixture models have been successfully used in video foreground/background segmentation (Stauffer and Grimson, 1999; Barnich and Van Droogenbroeck, 2010). Here, we exploit a similar approach that takes into account the image formation process. Here we propose a novel RG strategy based on an estimation which considers the image formation process (Calapez and Rosa, 2010) to define intrinsic properties of signal distribution in the image in question.

Our rationale is that confocal and 2-photon microscopy are based on sampling successive points in a focal plane to reproduce the spatial distribution of fluorescent probes within a sample. Hence, each pixel contains a discrete measure of the detected fluorescence within a sample interval, represented by a photon count, and certain amount of noise, deriving from different sources (Pawley, 2006; Calapez and Rosa, 2010). Therefore, statistical methods represent a natural way of describing confocal or 2-photon datasets. Different models have been proposed to depict confocal image properties (Calapez et al., 2002; Pawley, 2006). Specifically, mixture models (MM) have been suggested as the best descriptor of the sharp peaks and the long tails typical of background and low fluorescence distributions (Calapez and Rosa, 2010).

Given these considerations, we have developed a new Smart Region Growing algorithm (SmRG), which couples the RG procedure with a MM describing the signal statistics, to calculate local homogeneity predicates (i.e., local thresholds) for iteratively growing the structure to be segmented. Here, we describe the SmRG workflow for single-neuron segmentation. Then, we evaluate its performance in segmenting different neuron types from confocal and 2-photon datasets, comparing the results with those obtained with a gold standard manual reconstruction. Furthermore, we compare our algorithm with state-of-the-art (SoA) tools widely used in the field of neuron reconstruction.

## The Smart Region Growing (SmRG) Algorithm

### The Mixture Model

In its original version (Calapez and Rosa, 2010), the model is supposed to describe *K* different fluorescence levels or classes; the k-th class is described by the linear mixture model:

(1) $$\psi_{k}\,\left(y\right)=\alpha_{k}\,\psi_{B}\,\left(y-K_{0}\right)+\left(1-\alpha_{k}\right)\,\psi_{S\,k}\,\left(y-K_{0}\right)$$

where *y*, *K*_0_ and α_*k*_ denote the pixel intensity level, the system offset and the mixture parameter respectively. ψ_*B*_ is the distribution for the background pixels and is modeled according to a discrete normal distribution, with variance *v*_*B*_ and mean *K*_0_, and ψ_*Sk*_ is the intensity distribution of the k-th class pixels, described by a negative-binomial distribution with variance *v*_*Sk*_ and mean μ_*Sk*_. In accordance with (Calapez and Rosa, 2010) the negative-binomial distribution is re-parameterized in terms of

(2) $$p_{k}=\frac{\mu_{S\,k}}{v_{S\,k}}$$

and

(3) $$r_{k}=\frac{\mu_{S\,k}^{2}}{v_{S\,k}-\mu_{S\,k}}$$

For region growing purposes, it is reasonable to assume the presence of a single class *k* of pixels, at least locally. In this case, the complete model for a pixel *y*_*l*_ is described by the 5-parameter distribution:

(4) $$\begin{array}{c} \psi \,\left(y_{l};K_{0},v_{B},\alpha ,r,p\right)=\alpha \,\frac{1}{Z\,\left(v_{B}\right)}\,\exp\left(-\frac{{\left(y_{l}-K_{0}\right)}^{2}}{2\,v_{B}}\right)\\ \;+\left(1-\alpha \right)\,\frac{\Gamma \,\left(y_{l}-K_{0}+r\right)}{\left(y_{l}-K_{0}\right)!\,\Gamma \,\left(r\right)}\,p^{r}\,{\left(1-p\right)}^{y_{l}-K_{0}} \end{array}$$

where all the parameters are real values except for *K*_0_ which is an integer and α ∈ [0, 1].

The model fitting is done by means of an Expectation-Maximization (EM) algorithm in which:

1. *p* and *r* are obtained by the method of moments (eqs. 2, 3)
2. *K*_0_ and *v*_*B*_ are given by the maximization of the log-likelihood

(5) $$L\,\left(\Theta |Y,X\right)=\sum_{y=\min Y}^{\max Y}\ln\left(\psi \right)$$

3. α is given by the posterior density

(6) $$\alpha =\frac{\sum_{y=\min Y}^{\max Y}\alpha_{y}}{N}$$

### The Algorithm Outlined

The SmRG is an open-source algorithm developed in Matlab (The Mathworks-Inc., United States). A package of the functions needed for running the algorithm are available at http://www.centropiaggio.unipi.it/smrg-algorithm-smart-region-growing-3d-neuron-segmentation.

The SmRG is driven by a homogeneity predicate for establishing a local threshold based on the intensity levels of confocal datasets. Specifically, it exploits the statistics of the background and the signal distributions of the confocal acquisitions and a linear MM to determine the probability with which a given pixel (voxel) can be considered as part of the foreground or not, as described in section “The Mixture Model.” The rule to grow regions is then designed from these probabilities.

**FIGURE 1:**
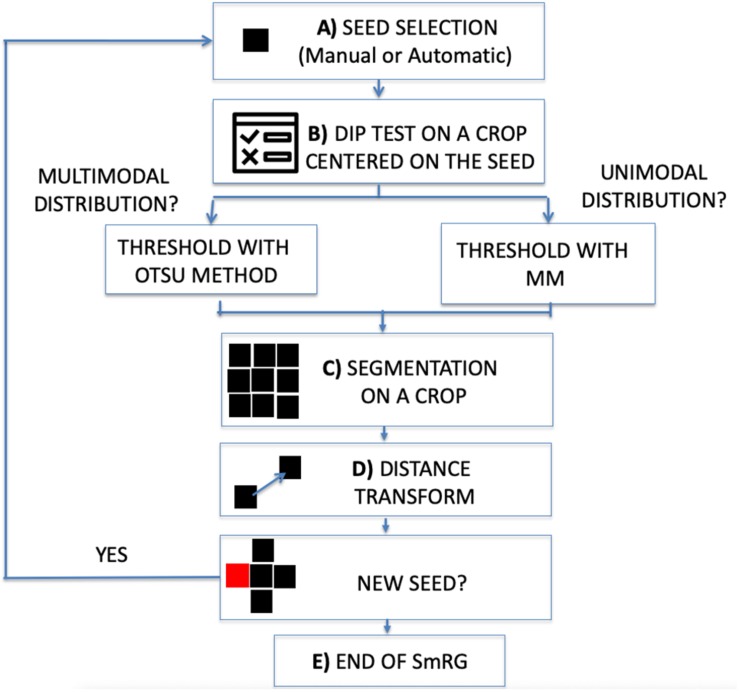
Workflow of the SmRG. **(A)** Manual or automatic seed selection. **(B)** Dip test to test for unimodality against multimodality on a MxNx3 crop centered on the seed. The threshold is determined with Otsu’s method or through the Mixture Model according to whether the distribution is multimodal or not. **(C)** 3D segmentation of a MxNx3 crop. **(D)** The regional maxima of the distance transform of the segmented MxNx3 crop are chosen as new seeds. **(E)** The procedure iterates until there are no more new seeds.

The workflow of the SmRG is sketched in Figure 1. It begins by selecting a seed, either manually or automatically (Figure 1A).

In the first case, the user is asked to identify the seed position by selecting a point on a focal plane (e.g., a pixel belonging to the soma), while in the latter the Hough transform (Nixon and Aguado, 2012) searches for spherical objects within the stack to identify the somata: the seed (or the seeds) is (or are) chosen as the center of the detected sphere (or spheres). Then, the homogeneity predicate is derived locally on an image volume centered on the seed. The volume dimension is a trade-off between the goodness-of-fit of the MM and the localness of the segmentation and by default is set to $\frac{N}{8}\times \frac{M}{8}\times 3$, where N and M are the on-plane size of the image stack. To ensure enough data points for MM fitting, the crop size is never smaller than 32×32×3. At this step a Hartigan’s dip test (Hartigan and Hartigan, 1986) (*p* < 0.01) is performed on the pixel intensity distribution of the crop to test for unimodality against multimodality (Figure 1B). In the case of multimodality the segmentation proceeds with Otsu’s method (Otsu, 1979), a well-known thresholding technique for multimodal distributions (Guo et al., 2012). Otherwise, a linear MM, considering the background as a normal distribution and the signal as a negative binomial, is fitted by means of an Expectation Maximization (EM) algorithm on the crop pixel intensity distribution. Indeed, mixture models combining normal and negative binomial distributions have been observed to fully characterize the signal associated with confocal images (Calapez and Rosa, 2010). The homogeneity predicate is derived from the posterior probability of the MM, α (or 1-α), denoting the probability at which a given pixel can be considered as part of the background (or the signal) distribution. The rule is thus obtained as a user defined threshold for α (e.g., with 1-α > 0.999 all the seed’s neighboring pixels whose probability of belonging to the signal exceeds 99.9% are segmented) (Figure 1C). Each pixel that satisfies this rule and is spatially connected to the seed within the crop is added to the object to be segmented. At this point, new seeds are chosen from the points just recognized as part of the neuron to be segmented. In particular, for each segmented plane the regional maxima of the distance transform (Maurer and Raghavan, 2003) are taken as new seeds (Figure 1D). The algorithm iterates for each detected seed and the process stops when there are no more pixels to add (Figure 1E).

**FIGURE 2:**
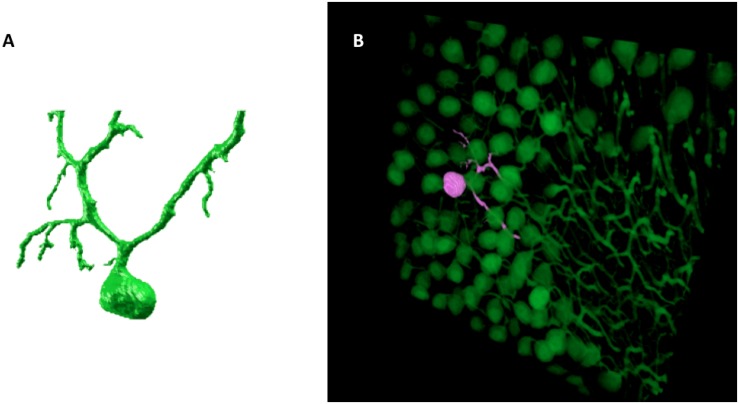
An example of SmRG outcome: **(A)** a Purkinje cell from clarified murine cerebellum acquired using a Nikon A1 confocal microscope; **(B)** the same Purkinje cells identified within its confocal dataset.

The result of the SmRG is a 3D matrix of logical values, whose true values represent the voxels constituting an isolated neuron. Figure 2 shows an example of a Purkinje cell segmented using the SmRG from a confocal dataset representing a 1 mm-thick slice from murine cerebellum, obtained after applying the CLARITY protocol described in Magliaro et al. (2016).

## Materials and Methods

To evaluate the SmRG’s performance, we processed two different sets of data. First, confocal acquisitions of 1 mm-thick slices of clarified cerebellum from a L7GFP mouse were analyzed to isolate Purkinje Cells (PCs) expressing Green Fluorescent Protein (GFP). The aim was to demonstrate (i) the SmRG’s accuracy with respect to a manual segmentation performed by experts, as it is still considered the gold standard for neuron segmentation (Al-Kofahi et al., 2003; Meijering, 2010), (ii) the SmRG’s reproducibility, and (iii) its ability to handle 3D microscopic datasets representing dense-packed neurons compared with other tools available in literature.

Then, Olfactory Projection (OP) Fibers dataset from the DIADEM challenge was processed with the SmRG. The SmRG reconstructions were quantitatively compared to the manually-traced gold-standards provided by the DIADEM. Moreover, 3D neuron segmentation was performed using other SoA tools evaluating the outputs against the DIADEM gold standards through the metrics SD, SSD and SSD%. This allowed an assessment of the SmRG’s ability to reconstruct 3D neuron morphology with the same precision and accuracy as SoA algorithms.

The tools used for both PC and OP datasets were the Vaa3D (version 3.200) app2 (Xiao and Peng, 2013), MST-tracing (Basu and Racoceanu, 2014), SIGEN (Ikeno et al., 2018) and MOST (Ming et al., 2013) plug-ins. They have been extensively validated in other reports and are widely used to compare reconstructions provided by new segmentation algorithms (Peng et al., 2014; Liu et al., 2016). A further quantitative comparative analysis with NeuroGPS (Quan et al., 2016) was performed was performed on the PC datasets.

### Datasets Representing PCs

#### Accuracy Test: SmRG Algorithm Versus Manual Segmentation

The confocal datasets representing dense-packed PCs from 1 mm-thick slices from clarified L7GFP murine cerebellum were those already manually segmented in Magliaro et al. (2017). They are available for download at http://www.centropiaggio.unipi.it/mansegtool. Specifically, *n* = 3 Purkinje cells from three different datasets were segmented automatically with the SmRG algorithm and manually by 6 experts with the ManSegTool, a tool purposely developed for facilitating the manual segmentation of 3D stacks (Magliaro et al., 2017). The matrix and voxel sizes for the three datasets are: (i) Dataset 1: 512 × 512 × 143, *x* = 0.62 μm/pixel, *y* = 0.62 μm/pixel, *z* = 1.24 μm/pixel; (ii) Dataset 2: 1024 × 1024 × 389, *x* = 0.31 μm/pixel, *y* = 0.31 μm/pixel, *z* = 0.62 μm/pixel (iii) Datasets3: 512 × 512 × 139, *x* = 0.62 μm/pixel, *y* = 0.62 μm/pixel, *z* = 1.24 μm/pixel.

The SmRG’s segmentation accuracy was evaluated by comparing morphometric features extracted from the two outputs. Briefly, we considered (i) the surface area, (ii) the volume, and (iii) the Sholl analysis (Sholl, 1955; Magliaro et al., 2017) of segmented structures. To compare Sholl profiles, we calculated the total area under the curve (AUC) using the trapezoidal rule thus obtaining a single measure for each profile (Binley et al., 2014). Statistical differences between the features in the manual segmented structures and those resulting from the SmRG were evaluated by means of the Friedman’s test with replicates. Friedman’s test allows testing treatments under study (i.e., columns) after adjusting for nuisance effects (i.e., rows). Replicates refer to more than one observation for each combination of factors. In our case, surface area, volume and the AUC of Sholl profiles were blocking factors (i.e., rows) with replicates represented by the three neurons, while users and SmRG represented treatments (i.e., columns). Thus, we are testing the null hypothesis of no difference between manual and SmRG-based segmentation.

#### SmRG Reproducibility

Reproducibility tests were performed by segmenting the same *n* = 3 PCs starting from different seeds. Specifically, we randomly chose 10 pixels picked from different regions of the neuron. Volume, surface area and AUC of Sholl profiles were obtained for each seed and the reproducibility was quantified for each neuron as the coefficient of variation of each measure (i.e., the standard deviation normalized by the mean).

#### SmRG vs. SoA Tools

In order to highlight the SmRG’s ability to segment single-neurons from confocal datasets represented densely-packed cells, we processed a 3D image stack with the App2, MST, SIGEN, MOST Vaa3d plugins and with NeuroGPS.

The reconstructions provided by the Vaa3D plugins and by SmRG were visually compared. On the other hand, *n* = 6 neurons were segmented with SmRG and NeuroGPS and manually through ManSegTool. After translating the volumetric information obtained with SmRG and ManSegTool in swc format, the three reconstructions were quantitatively compared by means of the following metrics: (i) the spatial distance (SD), (ii) the substantial spatial distance (SSD), and (iii) the percentual substantial spatial distance (%SSD). The spatial distance is estimated as it follows:

(7) $$S\,D=\frac{\sum_{i}d_{A\,B}\,\left(i\right)}{2\,\left|n_{A}\right|}+\frac{\sum_{j}d_{B\,A}\,\left(j\right)}{2\,\left|n_{B}\right|}$$

With

(8) $$d_{A\,B\,\left(i\right)}=a\,r\,g\,m\,i\,n_{j}\,\left|n_{A}\,\left(i\right)-n_{B}\,\left(j\right)\right|,j\in \left[1,\left|n_{B}\right|\right],i\in \left[1,\left|n_{A}\right|\right]$$

and

(9) $$d_{B\,A\,\left(j\right)}=a\,r\,g\,m\,i\,n_{i}\,\left|n_{B}\,\left(j\right)-n_{A}\,\left(i\right)\right|,i\in \left[1,\left|n_{A}\right|\right],j\in \left[1,\left|n_{B}\right|\right]$$

i.e., given two reconstructions, A and B, the spatial distance is obtained by averaging the Euclidean distance between the nodes of A and the nodes of B, i.e., *d*_*AB*_, with the reciprocal measure, i.e., *d*_*BA*_. Specifically, for each node belonging to A, *d*_*AB*_ is obtained by selecting the minimum distance between each node of B. *d*_*AB*_ is thus obtained by repeating this operation for every node of A and averaging the results. The same operation is performed with the nodes belonging to B, to obtain *d*_*BA*_.

The SSD is obtained by selecting the node pairs in A and B with a minimal distance above a given threshold S and then performing their average. Specifically, given:

(10) $$D_{A\,B}=\left\{d_{A\,B}\,\left(i\right)|d_{A\,B}\,\left(i\right)>S\right\}$$

and

(11) $$D_{B\,A}=\left\{d_{B\,A}\,\left(j\right)|d_{B\,A}\,\left(j\right)>S\right\}$$

Then, the SSD is defined as follows:

(12) $$S\,S\,D=\frac{\bar{D_{A\,B}}}{2}+\frac{\bar{D_{B\,A}}}{2}$$

Finally, the % SSD is obtained by estimating the ratio of nodes contributing to SSD. These metrics express the similarity of two different reconstructions (Peng et al., 2011b). Essentially, SD is a measure of how different two reconstructions are, while SSD and SSD% measure the extent of differences between two reconstructions considering only points above a tolerance threshold S. The tolerance threshold for the evaluation of the SSD metric was 2 (i.e., *S* = 2) voxels, as suggested in Peng et al. (2011a). Given that the SmRG’s output is a 3D logical matrix constituting the whole neuron, while the DIADEM gold-standard is a set of points of interest (i.e., a ^∗^.swc file), a thinning procedure was necessary to reduce the volumetric information in SmRG to a skeleton. To this end, we calculated the 3D skeleton of the SmRG output via a 3-D Medial Surface Axis Thinning Algorithm (Lee et al., 1994). From the points constituting the skeleton we reconstructed the corresponding ^∗^.swc file, ensuring a fair mapping between the DIADEM reference points and the SmRG ones.

Moreover, the precision, recall and F-score of the SmRG reconstructions were determined with respect to the gold-standard, quantifying the spatial overlap between the closest corresponding nodes of the two reconstructions (Powers, 2011) and varying the tolerance threshold from 0.5 to 5 voxels, to evaluate the SmRG’s sensitivity to this parameter (Radojeviæ and Meijering, 2018).

### DIADEM Datasets Representing OP Fibers

The dataset representing OP Fibers is available at http://diademchallenge.org/olfactory_projection_fibers_readme.html. It contains 9 separate drosophila olfactory axonal projection image stacks acquired with a two-photon microscope and their respective gold standard reconstructions provided by the DIADEM (Evers et al., 2005; Jefferis et al., 2007). We segmented all the neurons except OP2, since it contains many irrelevant structures (Liu et al., 2016). The SmRG and SoA algorithm reconstructions were compared with the DIADEM gold-standards. Comparisons between automatic tools were made by means of the metrics described in section “SmRG vs. SoA Tools.”

## Results

### Purkinje Cell (PC) Segmentation

#### SmRG vs. Manual Segmentation

**FIGURE 3:**
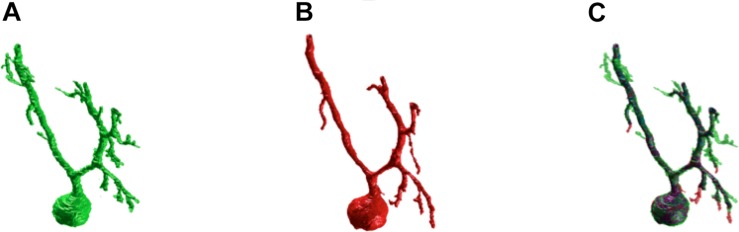
SmRG versus Manual Segmentation. **(A)** Gold-standard manual segmentation. **(B)** SmRG automatic segmentation. **(C)** Merge of manual (green) and automatic (red) segmentation, common voxels are reported in purple.

**FIGURE 4:**
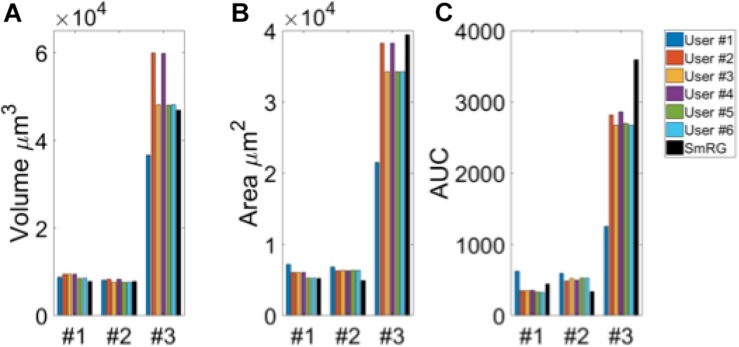
Testing SmRG accuracy **(A)** Neuron volume. **(B)** Neuron surface. **(C)** AUC (area under the curve) of Sholl profiles. Friedman’s test was performed with Volume, Area and AUC as blocking factors (rows, nuisance effects) with replicates (neurons #1, #2 and #3), and with users and SmRG as treatments (column). No statistical differences were observed (*p*-value = 0.8233).

**FIGURE 5:**
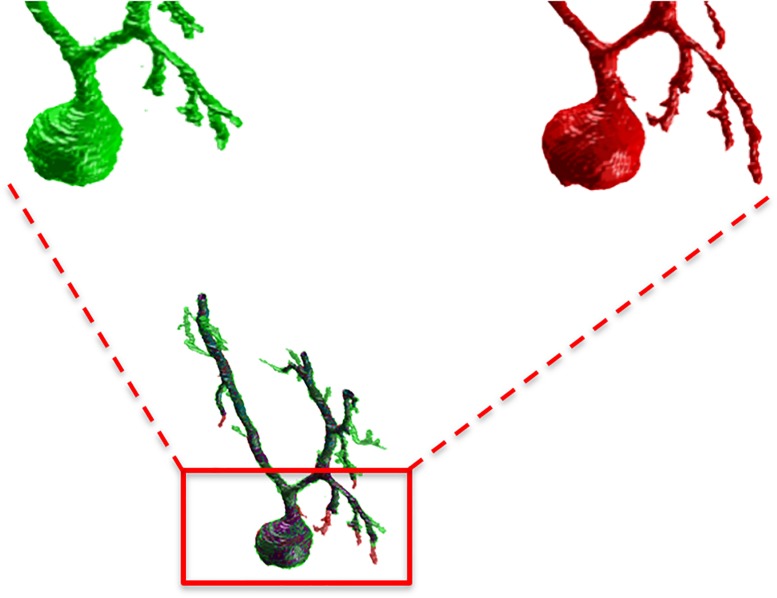
A detail of the manual and SmRG neuron reconstruction. It is clear that the SmRG segmentation (red) leads to a smoother volume than the manual (green) one.

Figure 3 shows an example of the same PC segmented by an expert and by the SmRG. The SmRG’s accuracy was assessed by comparing volume, surface area and AUC of Sholl profiles extracted from the segmented PCs with the results obtained by manually segmented ones (Figure 4). The single-neuron reconstructions provide quantitative information on the morphology of individual neurons in their native context where they are surrounded by neighboring cells. Clearly the algorithm developed is able to follow neurite arborization, segmenting smaller branches with similar performance to manual segmentation. Furthermore, the structure obtained with the SmRG is consistently characterized by a smooth volume, compared with the manual segmentation. A typical example is reported in Figure 5, showing a zoomed detail of manual and SmRG segmentation results.

The Friedman’s test showed no significant differences between the SmRG and the ManSegTool segmentation in terms of surface area, volume and Sholl profiles of the segmented structures (*p* = 0.8233); a detailed ANOVA table of the Friedman’s test is reported in Table 1. In summary, the results in the table demonstrate that the SmRG’s performance is comparable to that obtained from manual segmentation performed by experts in terms of the accuracy of the morphological parameters considered.

**TABLE 1:** Friedman’s ANOVA table.

Source	SS	Df	MS	Chi-sq	p>Chi-sq
Columns	110.94	6	10.4907	2.08	0.8233
Interaction	64.89	12	5.4074		
Error	2132.67	42	50.7778		
Total	2308.5	62			

*SS = Sum of Squares due to each sources; Df = Degree of freedom associated with each source; MS = Mean Squares, which is SS/Df; Chi-sq: Freedman Chi-square statistic; p: p-value for the Chi-square statistic.*

#### SmRG’s Reproducibility

Table 2 reports the coefficients of variation of volume, surface area and AUC of Sholl profiles for each segmented PC. The maximum coefficient of variation was equal to 0.0258, demonstrating the robustness of the SmRG to changes in initial conditions (i.e., the position of a seed belonging to the structure of interest).

**TABLE 2:** Results of SmRG’s reproducibility.

Neuron	Volume	Area	AUC
#1	0.0015	0.0025	7.8e−04
#2	0.0176	0.0258	0.0138
#3	0.0017	6.1e−04	0.0026

Coefficients of variation for neuron volume, area and AUC for n different RG seeds. *The coefficient of variation corresponds to the standard deviation divided by the mean σ/μ.

#### SmRG vs. Other Tools

**FIGURE 6:**
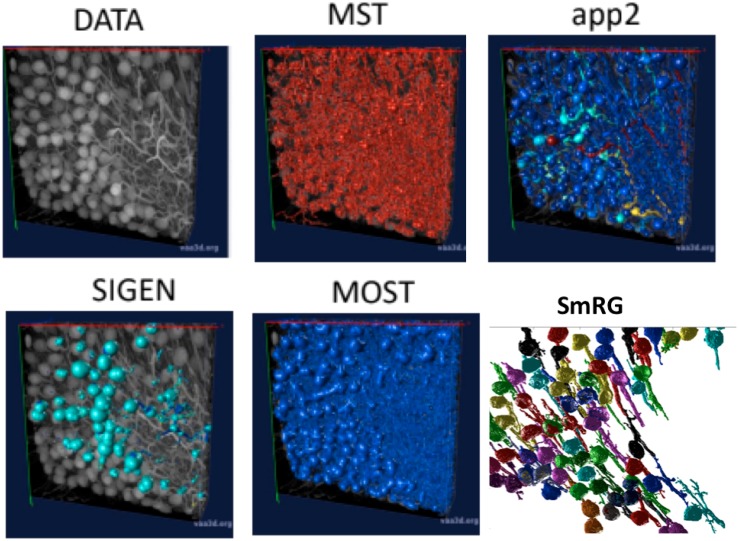
An example of a confocal dataset representing PCs from a clarified L7GFP murine cerebellum, segmented with MST, app2, MOST, SIGEN, and SmRG. None of the SOA tools is able to deal with this dense dataset, while the SmRG is able to isolate the PCs within the dataset. Different colors refer to the different neurons recognized.

Figure 6 shows an example of the outputs obtained segmenting the same confocal 3D stack with the App2, MST, SIGEN and MOST routines and with the SmRG. We were only able to assess the comparisons visually, since none of Vaa3D plugins was able to handle such dense datasets.

**FIGURE 7:**
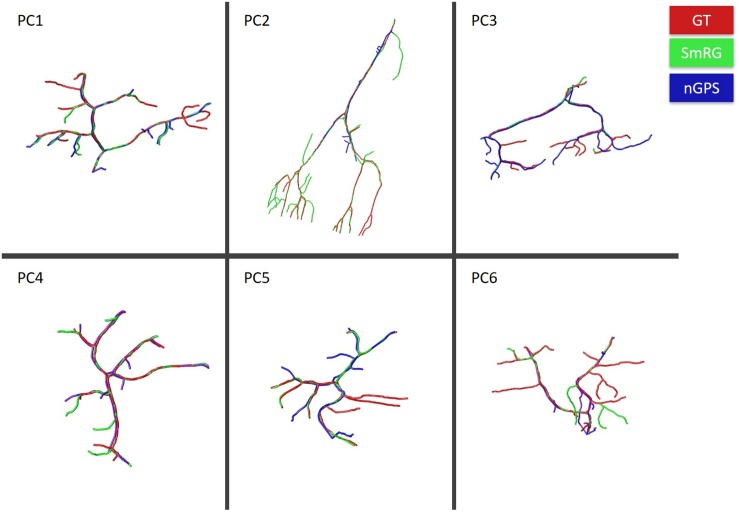
PCs segmented with SmRG (green) and NeuronGPS (blue) and compared with the manually segmented gold-standard (red).

**FIGURE 8:**
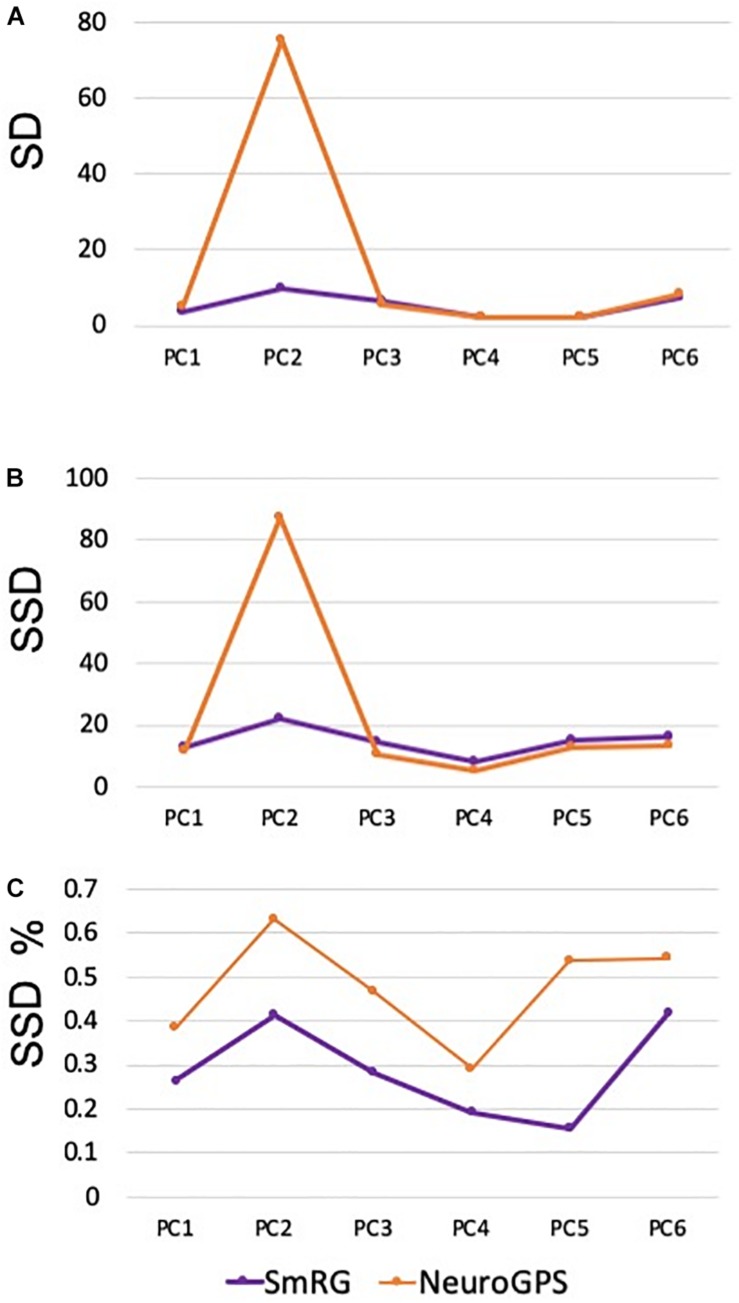
Accuracy of SmRG and NeuronGPS against the manually segmented gold standard for different PCs. **(A)** SD **(B)** SSD, and **(C)** percentage SSD.

**FIGURE 9:**
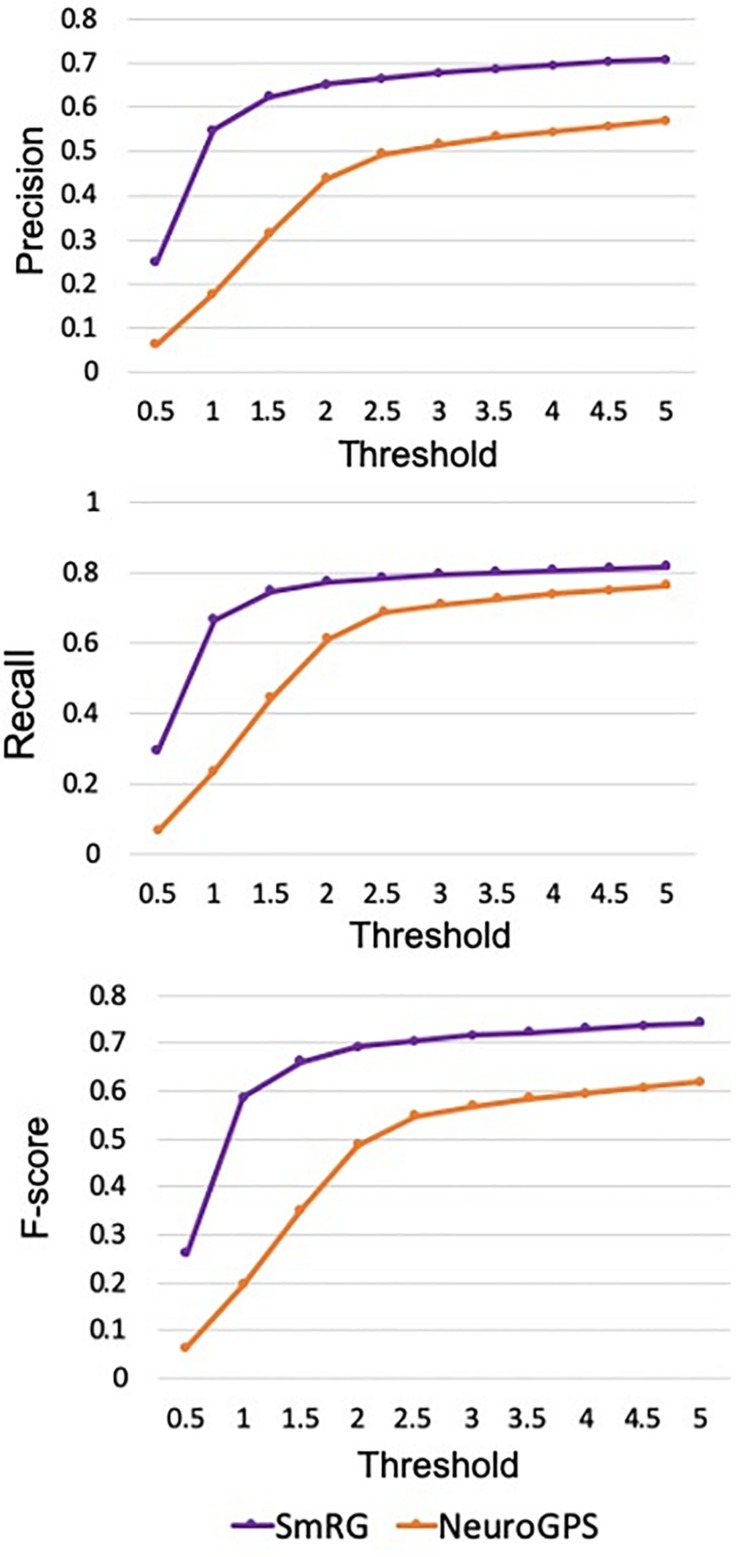
Precision, Recall and F-Score for varying thresholds of SSD evaluation. SmRG has always better performance than NeuronGPS for increasing values of the threshold.

Figure 7 reports the same dense packed PCs segmented with both SmRG and NeuroGPS, showing that the performance of the two tools is comparable. This is also evident from the SD, SSD and SSD% metrics obtained with respect to the gold standard provided by the manual segmentation for all the neurons segmented except for PC2 (Figure 8). Moreover, the average precision, recall and *F*-score in Figure 9 shows better precision and accuracy for our tool with respect to NeuroGPS for *S* = 2.

### OP Fibers: SmRG vs. the DIADEM Gold-Standard

**FIGURE 10:**
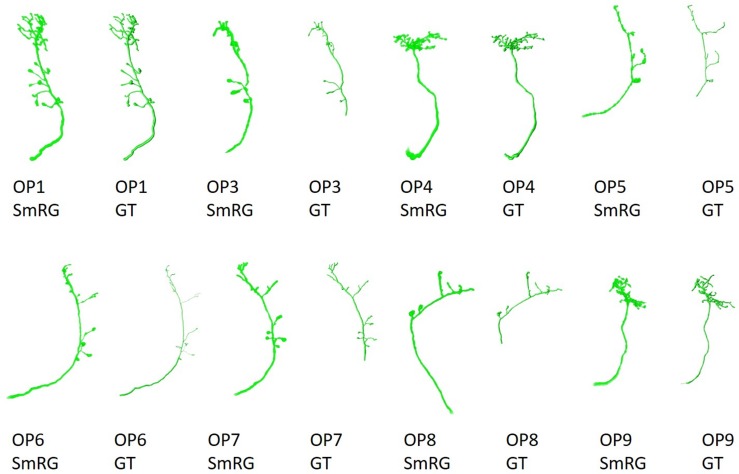
OP fibers segmented with SmRG and compared with the DIADEM gold-standard (GS). Please note that for OP3, OP5, OP7, and OP8 the gold standard reconstruction misses some terminal branches (see DIADEM FAQ at http://diademchallenge.org/faq.html).

Olfactory Projection fibers segmented with the SmRG are reported in Figure 10, along with the manually-traced gold-standard provided by the DIADEM.

One of the distinctive characteristics of the SmRG is its ability to trace the axon topology of OP fibers while maintaining 3D volumetric information on neurons and their arbors. Indeed, the structure obtained with the SmRG is a smooth three-dimensional volume with voxel-resolution details on neuron morphology; a feature not available from swc structures. As a consequence, the SmRG reconstructions in Figure 10 appear thicker than the 3D rendering of ^∗^.swc gold-standards.

As evident from the figure, some terminal branches of OP fibers are not comprised in the manually traced gold standard, since they have no effect on DIADEM metrics (Brown et al., 2011; Gillette et al., 2011). Nonetheless, the SD, SSD, and SSD% metrics used in this work are naturally biased by these missing branches. Thus, the comparison between automatic reconstructions and gold standard were limited to those branches included in by the DIADEM gold standard.

**FIGURE 11:**
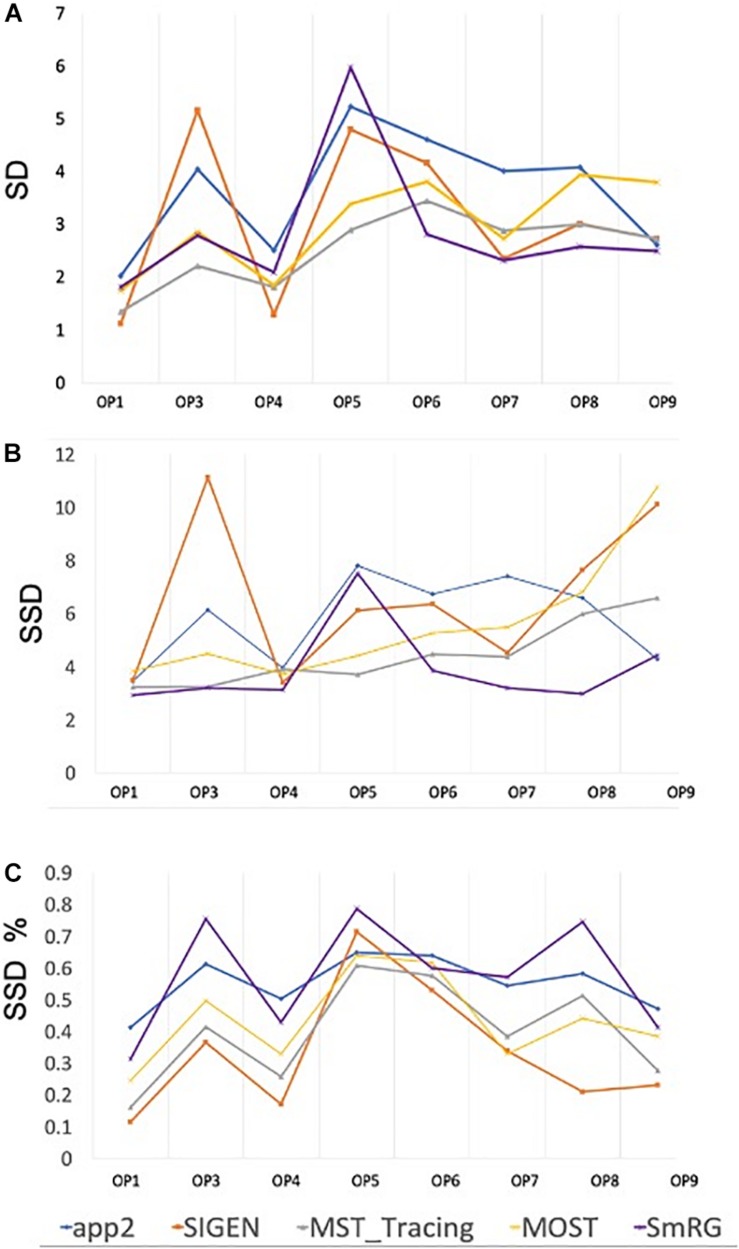
Accuracy of SmRG and SoA tools against the DIADEM gold standard for different OP fibers. **(A)** SD **(B)** SSD, and **(C)** percentage SSD.

When evaluated against other SoA tools, the SmRG was observed to be comparable in terms of SD. On the other hand, our algorithm achieved the lowest values of SSD among all tools considered (with the exception of segmentation of OP5). It should be noted that the value of SSD% was higher for the SmRG with respect to other algorithms, since the estimation of the skeleton from the 3D output of SmRG produced a higher number of nodes compared to the other methods (Figure 11).

**FIGURE 12:**
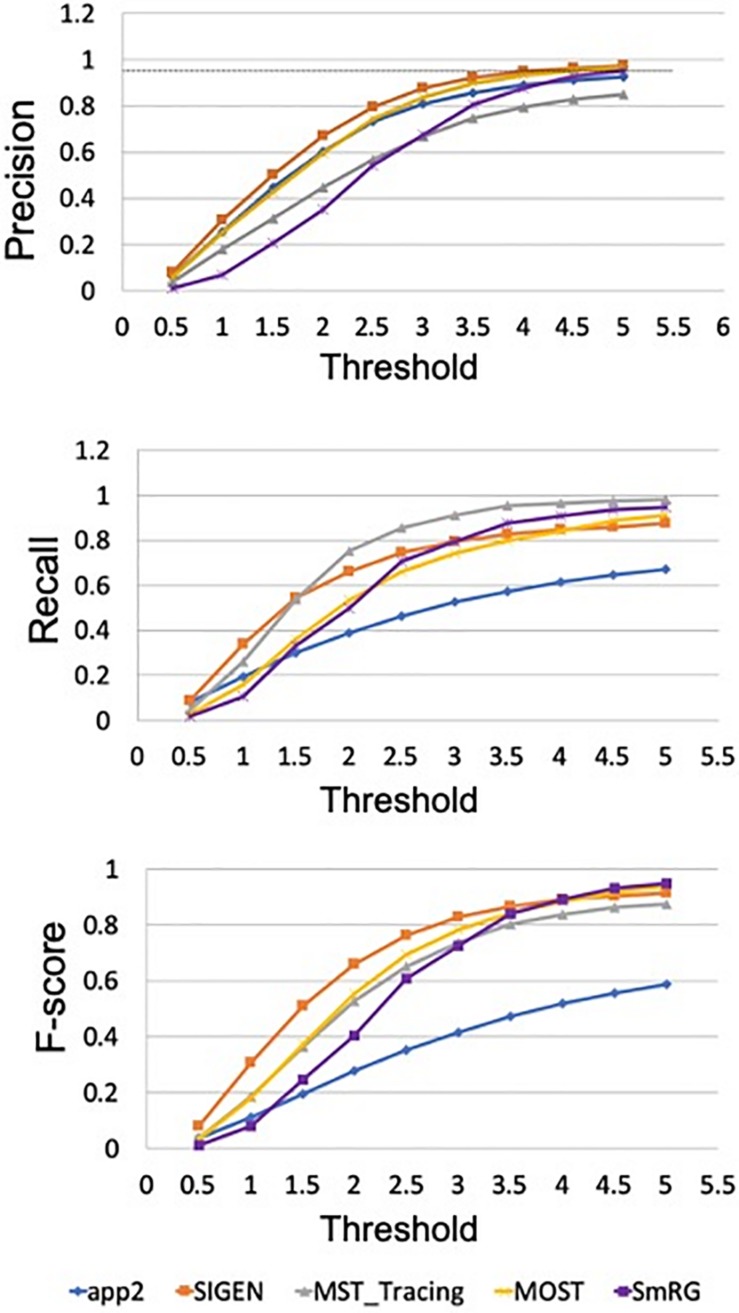
Precision, Recall and F-Score for varying thresholds of SSD evaluation. SmRG and SoA tools have similar performance for increasing values of the threshold. For thresholds greater than four voxels, SmRG has the highest *F*-Score. For *S* = 5, we obtained *P* = 0.9538 ± 0.0350, *R* = 0.9770 ± 0.0183 and *F* = 0.9651 ± 0.0248 (mean ± st. deviation) for the SmRG.

In Figure 12 the average precision, recall and F-score across OP fibers are reported for SmRG and SoA tools as a function of the value of S. For *S* = 5, the SmRG outperforms other tools in terms of F-score which highlights its ability to segment OP fibers with high accuracy.

## Discussion

The SmRG for the automatic segmentation of microscopic data exploits the signal statistics typical of confocal and 2-photon images (Calapez and Rosa, 2010). Datasets representing neural tissues from different species, processed using different protocols (i.e., clarified murine cerebella and Drosophila brains fixed using classical procedures) and acquired with different imaging tools (i.e., confocal and two photon microscopy) were used to test the algorithm. The goodness of the SmRG reconstruction was compared with manually traced gold-standards as well as with algorithms available in the SoA.

A quantitative analysis of the SmRG’s accuracy with PC datasets was performed for three different neurons, whose manually segmented counterpart was available in Magliaro et al. (2017). Although a limited set of neurons were analyzed, the reconstructions of the SmRG and the manually-segmented gold standards were comparable; moreover, the seeding and RG procedure was shown to be robust and independent of initial conditions. The analysis performed on PCs from clarified tissues highlighted the efficacy of the algorithm developed in isolating single neurons from densely-packed data with respect to some of the most widely used single neuron reconstruction tools available in the SoA (i.e., app2, MOST, MST-tracing, SIGEN) (Ming et al., 2013; Xiao and Peng, 2013; Basu and Racoceanu, 2014; Ikeno et al., 2018). In particular, none of the Vaa3D plug-ins allowed the reconstruction of 3D neuron morphology from the confocal stacks representing neurons in their native 3D context, limiting the evaluation of the SmRG’s performance to a visual comparison. Indeed, many SoA algorithms perform extraordinarily well with low-quality images possessing noisy points, large gaps between neurites and non-smooth surfaces (Liu et al., 2016), since they were likely developed specifically for such purposes. On the contrary, they may perform modestly or even fail in reconstructing densely-packed neurons (Hernandez et al., 2018), such as PCs in the murine cerebella because the images have very different properties (i.e., a large number of pixels with high intensities). The quantitative analyses of SmRG and NeuronGPS’ outcomes showed comparable performance of the two tools in terms of reconstructed arbors. In particular, SSD and SSD% values were similar for all PCs except for PC2, in which SmRG performs drastically better than NeuronGPS. Interestingly, SmRG reached a better precision (P) and accuracy (F-score) for all used thresholds with respect to NeuronGPS.

Reconstructions of OP fibers from the DIADEM challenge resulted in a comparable performance between the SmRG and well-established tools for neuron reconstruction in terms of SD, SSD, and SSD%. Specifically, the algorithm proposed here outperformed other tools in terms of SSD, which quantifies the discrepancy between two outcomes (Peng et al., 2011a), in almost all reconstructions. On the other hand, the SmRG exhibited higher values in the SSD% score. It should be noted that the gold-standard OP reconstructions are available in.swc format. Therefore, in order to compare the volumetric SmRG’s outputs with the gold standards, firstly we were forced to reduce the information by means of a thinning algorithm. The thinning algorithm inevitably introduces mismatches, since it depends on the 3D morphology of the neuron, thus biasing the meaningfulness of the SSD% values when comparing SmrG and SoA tools (Liu et al., 2016). The precision and recall of SmRG outcomes with respect to the manually traced gold-standard provided by the DIADEM highlighted the performance of our tool with respect to SoA algorithms in the segmentation of OP fibers). In particular, for the highest values of the tolerance threshold considered, the SmRG’s average values of precision, recall and f-score were all above 95%. This suggests that, although the algorithm was developed for segmenting neurons from clarified cerebral tissue, segmentation procedures based on local signal and noise statistics may be a successful strategy for “single-neuron” settings, and thus for delivering an adaptive and generalized algorithm, applicable to different contexts.

When two neurons naturally touch each other and the signal intensity is high, SmRG may reconstruct the two objects as one, thus requiring their post-splitting. A watershed-based routine for separating neurons is provided at http://www.centropiaggio.unipi.it/smrg-algorithm-smart-region-growing-3d-neuron-segmentation. Nevertheless, we also take advantage of the lower intensity values of neuron boundaries with respect to neuron bodies. This heterogeneity in pixel intensity is exploited in SmRG and quantified by the mixture parameter. As a result, neuron boundaries with lower intensity values are not segmented, controlling for possible false merge errors.

We would like to highlight that SmRG was not compared with SoA segmentation approaches in terms of computational times. Indeed, tools such as app2, MST, SIGEN, MOST and NeuroGPS outperform our algorithm as they provide faster segmentations. However, while the Vaa3D plugins provide 3D neuron reconstructions with comparable accuracy and precision (Figure 8) for sparsely labeled data, they fail when performing segmentations of densely-packed neurons. As regards the tool described by Quan et al. (2016), the strength of SmRG lies in the amount of morphological information it provides with respect to the NeuroGPS neuron tracing.

## Conclusion

Despite the numerous attempts addressed at 3D neuron reconstruction, little attention has been paid to delivering automatic and robust methods capable of dealing with the large variability of datasets representing densely-packed neurons, as well as for digitizing the morphology and volumetric characteristics of the segmented structures. As a result, the majority of algorithms are only able to handle with sparsely labeled data, compelling neuroscientists to manually segment images representing intricate neuronal arborisations and to reducing 3D space-filling neurons to skeletonized representations.

The SmRG, an open-source Matlab-based algorithm for the segmentation of complex structures in 3D confocal or 2-photon image stacks, overcome these setbacks. It provides an accurate reconstruction of 3D neuronal morphology acquired using confocal microscopy, which accounts for 80% of user needs in imaging facilities. The SmRG can potentially be extended to other imaging modalities (e.g., super-resolution microscopy) adopting the same statistical framework for identifying the signal and noise distribution from 3D images.

In addition, our tool allows the extraction of several useful morphological features from the segmented neurons. Preserving the volumetric information is an essential step for deciphering the Connectome. Besides structural mapping, from a biological perspective, digital 3D neuron reconstruction is crucial for the quantitative characterization of cell type by morphology and the correlation between morphometric features and genes (e.g., between wild-type and model animals) or patho-physiology (e.g., the detection of neuronal morphological anomalies in diseased individuals compared to healthy ones) (Acciai et al., 2016).

Future improvements could be obtained by coupling the NeuroGPS method (Quan et al., 2016) which rely on human strategies to separate individual neurons, with the SmRG’s one, thus leveraging on both the geometric constraints of the former and the statistical properties of the latter, taking the best from both the approaches.

In conclusion, the SmRG can facilitate the identification of the different neural types populating the brain, providing an unprecedented set of morphological information and new impetus toward connectomic mapping.

## Data Availability Statement

Publicly available datasets were analyzed in this study. This data can be found here: http://diademchallenge.org/data_set_downloads.html, http://www.centropiaggio.unipi.it/mansegtool.

## Author Contributions

AC, CM, and NV: conception and design of the segmentation tool. AC: implementation and testing of the algorithm. All authors interpretation of data and drafting of the manuscript.

## Conflict of Interest

The authors declare that the research was conducted in the absence of any commercial or financial relationships that could be construed as a potential conflict of interest.
